# Age-Specific Imbalance of Circulating Tfh Cell Subsets and Its Association With Gout-Targeted Kidney Impairment

**DOI:** 10.3389/fimmu.2020.625458

**Published:** 2021-01-11

**Authors:** Yefei Huang, Xinyu Wu, Lian Gui, Yutong Jiang, Liudan Tu, Xiaomin Li, Boxiong Jiang, Yina Wang, Xuqi Zheng, Qiujing Wei, Qiuxia Li, Jiayong Ou, Zena Chen, Ya Xie, Zhiming Lin, Zetao Liao, Linkai Fang, Minli Qiu, Shuangyan Cao, Jieruo Gu

**Affiliations:** ^1^ Department of Rheumatology, The Third Affiliated Hospital of Sun Yat-Sen University, Guangzhou, China; ^2^ Medical Examination Center, The Third Affiliated Hospital of Sun Yat-Sen University, Guangzhou, China; ^3^ VIP Medical Center, The Third Affiliated Hospital of Sun Yat-Sen University, Guangzhou, China

**Keywords:** gout, Tfh1/2 cells, aging, B cells, kidney impairment

## Abstract

**Objective:**

Gout is a chronic disease characterized by the deposition of monosodium urate (MSU) crystals in tissue. Study with a focus on adaptive immune response remains to be understood although innate immune response has been reported extensively in gout etiology. Our study attempted to investigate the association of gout-related immune cell imbalance with clinical features and comorbidity with renal impairment and the implicated pathogenesis *via* the assessment of T and B cell subsets in different activity phases or with immune effects combined with the analyses of clinical parameters.

**Methods:**

Fifty-eight gout patients and 56 age- and sex-matched healthy individuals were enrolled. To learn the roles of circulating T cells, a lymphocyte profile incorporating 32 T cell subsets was tested from isolated freshly peripheral blood monocyte cells (PBMCs) with multiple-color flow cytometry. Furthermore, the collected clinical features of participants were used to analyze the characteristics of these differential cell subsets. Stratified on the basis of the level of creatinine (Cr, enzymatic method), all patients were categorized into Cr^low^ (Cr ≤ 116 μmol/L) and Cr^hi^ (Cr > 116 μmol/L) groups to exploit whether these gout-associated T cell subsets were functional in gout-targeted kidney dysfunction. The differentiation of B cells was investigated in gout patients.

**Results:**

Our results show that CD 4^+^ T cells, Th2 cells, and Tc2 cells were upregulated, whereas Tc17 cells were downregulated. Tfh cells skewed toward the polarization of Tfh2 cells. Specifically, Tfh2 cells increased, but Tfh1 cells decreased, accompanied with aging for gout patients, suggesting that age might trigger the skewing of Tfh1/Tfh2 cell subsets to influence gout development. Moreover, Tfh2 cells were connected to renal dysfunction as well. No alterations of B cell subsets were observed in patients when compared to controls.

**Conclusions:**

Our data demonstrate age-specific dysfunctions of Tfh1/2 cells in gout occurrence, and Tfh2 cell upregulation is associated with gout-targeted renal dysfunction. However, Tfh2 cells may function in auto-inflammatory gout independent of helping B differentiation, and an in-depth study remains to be conducted.

## Introduction

Gout is a systemic disease caused by the deposition of monosodium urate (MSU) crystals in tissue (1), characterized most commonly by episodes of acute and extremely painful arthritis, normally impairing the big toe but involving other joints in some patients as well (2). Previous studies indicate that an MSU crystal–mediated cytoplasmic NOD-like receptor family pyrin domain containing 3 (NALP3) inflammasome pathway in the innate immune system exists in the progression of gouty inflammation (3, 4). Recently, some specific T cell populations were also reported to participate in adaptive immune responses in gouty arthritis. Lee et al. found that the receptor activator of nuclear factor-κ B ligand (RANKL)-expressed T cells destroy the bone to some degree in gout arthritis (5). The differentiation and activation of Th17 cells was induced by MSU crystals in the presence of IL-1α/β and IL-18 (6). However, to our knowledge, only a few investigations have exploited the roles of different T lymphocyte subsets in the predisposition to gout.

Gout attacks usually begin, ranging from the fourth to the sixth decade. A large epidemiological study in 2005 shows that the prevalence is about 1% in men aged within a range of 35–44 years and increases to more than 7% in those over 65 years (7). This evidence suggests that gout prevalence depends highly on age. Compelling proof identifies that aging causes the decline of the innate and adaptive immune systems, which is also called immunosenescence. Age-associated defects on T cell function and phenotypes are reported (8, 9). However, the association of aging with T cell function remains be delineated in gout.

In this study, we attempt to investigate gout-related immunophenotyping alterations and their associations with age and the comorbidity with renal dysfunction with flow cytometry in extensive lymphocyte profiles.

## Methods

### Ethics Statement

Ethics approval for this study was permitted by the medical ethics committee of the Third Affiliated Hospital of Sun Yat-sen University in compliance with the Declaration of Helsinki. The written informed consents were obtained from all participants for research use.

### Study Subjects

Cases enrolled were outpatients and inpatients with gout registered in the rheumatology division of the Third Affiliated Hospital of Sun Yat-sen University. Healthy participants were enrolled from the medical examination center of the hospital. Gout patients were identified according to the American College of Rheumatology 1977 diagnosis criteria for gout. Participants aged ≤44 years were defined as young, and those >44 years old were defined as middle-aged and elderly according to World Health Organization criteria. Controls had no inflammatory or rheumatic diseases or cancers and no antibiotic or immune-suppressing medicine use. Gout participants were excluded if they had other rheumatic disorders, inflammatory diseases, or cancers. Clinical features were collected, including demographics, creatinine (Cr), and estimated glomerular filtration rate (eGFR).

### Lymphocyte Immunophenotyping by Flow Cytometry

Immunophenotyping of peripheral blood lymphocytes was quantified with flow cytometry (BD) according to the manufacturer’s instructions. Peripheral blood mononuclear cells (PBMCs) from freshly collected EDTA-anticoagulated whole blood were isolated, followed by incubation and tests with a panel of the following individual or combinations of labeled antibodies encompassing CD3-PerCP-Cy5.5, CD4-APC-H7, CD8-BV510, CD127-BV421, CD25 L-PE, CD45RAL-FITC, CCR7-AF647, and CD28-PE-Cy7 for T cell subsets; CD3-APC-H7, CD4-PE-Cy7, CD8-PerCP-Cy5.5, CD183-AF488, CD196 (CCR6)-BV510, CXCR5 (CD185)-AF647, CD194-BV421, and CD279L-PE for T cell subsets; and CD45-APC-H7, CD19-PerCP-Cy5.5, CD27-BV421, IgD-BB515, IgM-BV510, CD38L-APC, CD24L-PE, and CD21-PE-Cy7 for B cell subsets or isotype-matched controls for 30 min. More detailed information about T/B cell labels for categorization are described in **Table 1**. For intracellular markers, fixed and targeted cells were fixed, permeabilized, and then stained using Alexa Fluor 488-anti-PD-1 (BD) to analyze. Finally, the cells were suspended with phosphate-buffered saline and then analyzed with BD flow cytometry.

**Table 1:** The alterations of T cell profile embodied in gout.

Parameters	Marker	HCs (*n*=56)	Gout (*n*=58)	*P* value
Age (years, median (P25, P75))		43.500 (32.250,53.000)	47.000 (38.750,54.250)	0.078
Sex ratio, % of male		98.2	98.3	0.980
T cells (%, median (P25, P75))	CD3^+^	68.25(61.075,73.175)	68.8(63.15,77.225)	0.159
CD4^+^ T cells (%, mean ± SD)	CD3^+^CD4^+^	51.729 ± 14.9052	60.607 ± 15.5475	**0.002**
CD8^+^ T cells (%, median (P25, P75))	CD3^+^CD8^+^	28.2(22.05,39.525)	28.5(21.15,37.325)	0.838
Double positive T cells (%, median (P25, P75))	CD3^+^CD4^+^CD8^+^	1.14(0.7625,2.15)	1.065(0.695,1.47)	0.308
Naïve CD4^+^ T cells (%, median (P25, P75))	CD3^+^CD4^+^CD45RA^+^CCR7^+^	20.1(10,28.25)	17.15(13.1,30.325)	0.975
Terminally differentiated CD4^+^ T cells (%, median (P25, P75))	CD3^+^CD4^+^CD45RA^+^CCR7^-^	18.9(11.625,33.75)	14.9(9.265,28.05)	0.136
Central memory CD4^+^ T cells (%, median (P25, P75))	CD3^+^CD4^+^CD45RA^-^CCR7^+^	9.22(4.875,13.425)	9.905(4.99,14.55)	0.638
Effector memory CD4^+^ T cells (%, mean ± SD)	CD3^+^CD4^+^CD45RA^-^CCR7^-^	45.995 ± 11.6895	50.176 ± 13.8890	0.085
Exhausted CD4^+^ T cells (%, median (P25, P75))	CD3^+^CD4^+^CD28^-^	2.65(1.145,6.9325	2.825(1.0425,5.03)	0.725
Functional CD4^+^ T cells (%, median (P25, P75))	CD3^+^CD4^+^CD28^+^	97.3(93.1,98.875)	97.2(94.975,98.925)	0.755
Regulatory T cells (%, median (P25, P75))	CD3^+^CD4^+^CD25^+^CD127^-^	3.935(2.94,9.8325)	3.02(1.91,7.0625)	0.059
Naïve CD8^+^ T cells (%, median (P25, P75))	CD3^+^CD8^+^CD45RA^+^ CCR7^+^	13.75(7.73,23.475)	13.45(5.7675,20.5)	0.479
Terminally differentiated CD8^+^ T cells (%, median (P25, P75))	CD3^+^CD8^+^CD45RA^+^ CCR7^-^	46.05(31.9,63.525)	48.1(35.65,58.775)	0.816
Central memory CD8^+^ T cells (%, median (P25, P75))	CD3^+^CD8^+^CD45RA^-^ CCR7^+^	0.785(0.4975,1.3625)	0.665(0.475,1.685)	0.604
Effector memory CD8^+^ T cells (%, median (P25, P75))	CD3^+^CD8^+^CD45RA^-^ CCR7^-^	32.25(24.9,44.6)	34.55(24.05,47.025)	0.783
Exhausted CD8^+^ T cells (%, median (P25, P75))	CD3^+^CD8^+^CD28^-^	33.85(11.225,49.2)	25.8(7.155,44.525)	0.199
Inactive virus-specific CD8^+^ T cells (%, median (P25, P75))	CD3^+^CD8^+^CCR7^-^CD45RA^-^ CD127^hi^	70.5(53.5,89.575)	68.6(56.1,81.175)	0.559
Inactive and terminally differentiated virus-specific CD8^+^ T cells (%, median (P25, P75))	CD3^+^CD8^+^CCR7^-^CD45RA^+^ CD127^hi^	67.15(25.325,90.2)	44.05(23.725,65.425)	0.051
Active virus-specific CD8^+^ T cells (%, median (P25, P75))	CD3^+^CD8^+^CCR7^-^CD45RA^-^ CD127^lo^	29.5(10.395,46.5)	31.4(18.825,43.9)	0.557
Active and terminally differentiated virus-specific CD8^+^ T cells (%, median (P25, P75))	CD3^+^CD8^+^CCR7^-^CD45RA^+^ CD127^lo^	32.85(9.84,74.675)	55.95(34.575,76.275)	0.051
**Helper T cell subsets**				
Tfh (%, median (P25, P75))	CD3^+^CD4^+^CXCR5^+^	19.5(14.4,23.65)	20.75(17.225,25.625)	0.107
Th1 (%, median (P25, P75))	CD3^+^CD4^+^CXCR5^-^CXCR3^+^CCR4^-^	14.55(10.425,20)	14.95(10.875,20.425)	0.892
Th2 (%, median (P25, P75))	CD3^+^CD4^+^CXCR5^-^CXCR3^-^CCR4^+^	13.85(10.8,18.4)	16.7(13.325,22.45)	**0.002**
Th17 (%, median (P25, P75))	CD3^+^CD4^+^CXCR5^-^CXCR3^-^CCR4^-^CCR6^+^	1.545(0.71,2.7625)	1.69(0.8425,3.1825)	0.542
Tfh1 (%, median (P25, P75))	CD3^+^CD4^+^CXCR5^+^CXCR3^+^CCR4^-^	11.7(10.1,14.675)	10.7(9.2525,12.6)	**0.038**
Tfh2 (%, mean ± SD)	CD3^+^CD4^+^CXCR5^+^CXCR3^-^CCR4^+^	35.807 ± 7.3297	41.238 ± 7.8087	**0.000**
Tfh17 (%, median (P25, P75))	CD3^+^CD4^+^CXCR5^+^CXCR3^-^CCR4^-^CCR6^+^	7.14(5.08,12.175)	7.745(5.4125,9.925)	0.892
Tc1 (%, median (P25, P75))	CD3^+^CD8^+^CXCR5^-^CXCR3^+^CCR4^-^	47.6(31.4,58.575)	41.8(27.4,54.275)	0.256
Tc2 (%, median (P25, P75))	CD3^+^CD8^+^CXCR5^-^CXCR3^-^CCR4^+^	2.35(1.3275,4.1675)	2.955(2.2825,4.46)	**0.038**
Tc17 (%, median (P25, P75))	CD3^+^CD8^+^CXCR5^-^CXCR3^-^CCR4^-^CCR6^+^	4.74(2.04,7.01)	2.445(1.33,4.735)	**0.018**
Peripheral Th (%, mean ± SD)	CD3^+^CD4^+^CXCR5^-^PD-1^+^	57.889 ± 6.6860	58.321 ± 4.7455	0.691
Activated Tfh (%, median (P25, P75))	CD3^+^CD4^+^CXCR5^+^PD-1^+^	15.65(12.55,17.675)	17.05(13.1,20.3)	0.067

Bold values denote p < 0.05.

### Statistical Analysis

SPSS 23.0 software was used for statistical analysis. The Shapiro–Wilk test was used for distribution. Reference ranges were calculated using mean ± standard deviation for parametric data and median (P25, P75) for nonparametric data. The distinction between two groups was compared using 2-sided tests for parametric data and nonparametric Wilcoxon rank sum tests. Gender ratio was compared between controls and patients using a chi-square test. Multiple group comparisons were conducted by one-way ANOVA, followed by LSD analysis of variance. Association of variables with age and renal function parameters were tested using a parametric Pearson test or nonparametric Spearman’s rank correlation test. *P*<0.05 was considered as statistical significance.

## Results

### Some Specifically Changeable T Lymphocytes Were Observed in Gout Patients

In the study, 58 gout cases and 56 healthy gender- and age-matched participants were enrolled for the assessment of human immune cell compartmentalization. The Chi-square test showed no distinctions for gender ratio between the case and control groups. To evaluate comprehensively the functional subpopulations of T lymphocytes in gout status, 32 T cell subsets were measured (**Table 1**). Median (P25, P75) used for mean age was 43.50 (32.25, 53.00) years in the healthy control (HC) group and 47.00 (38.75, 54.25) years in gout patients. When compared to the healthy cohort, five parameters containing CD3^+^CD4^+^ T cell percentage (*p*=0.002), and CD3^+^CD4^+^CXCR5^-^CXCR3^-^CCR4^+^ Th2 cell percentage (*p*=0.002), CD3^+^CD4^+^CXCR5^+^CXCR3^-^CCR4^+^ Tfh2 cell percentage (*p*=0.000) and CD3^+^CD8^+^CXCR5^-^CXCR3^-^CCR4^+^ Tc2 (*p*=0.038) were overexpressed and CD3^+^CD4^+^CXCR5^+^CXCR3^+^CCR4^-^ Tfh1 (*P*=0.038) cells and CD3^+^CD8^+^CXCR5^-^CXCR3^-^CCR4^-^CCR6^+^ Tc17 (*P*=0.018) were underexpressed significantly in gout populations. Other T lymphocytes had no significant alterations. This evidence implies that the frequencies of some lymphocyte subsets were dysregulated in a gout attack.

### The Dysregulation of Tfh1/Tfh2 Cell Subsets Is Age-Specific

**Figure 1:**
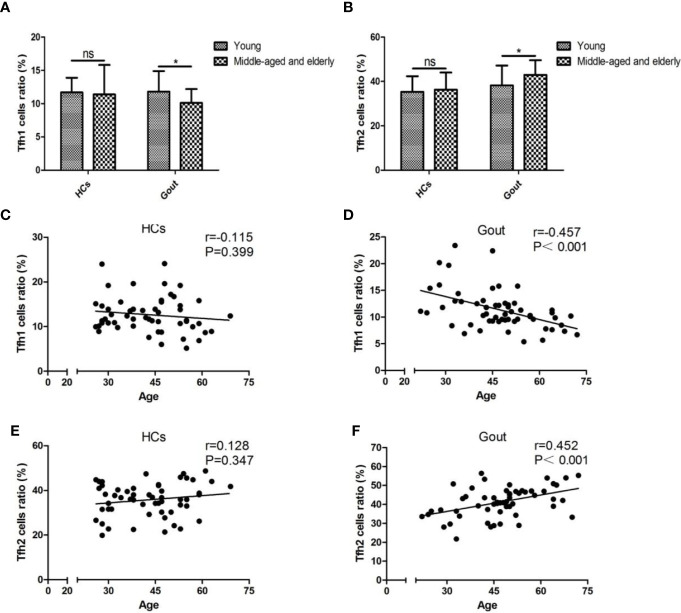
Tfh1/Tfh2 cell subsets are age-specific in gout patients. **(A, B)** The abundances of Tfh1 and Tfh2 cells are compared by *t* test for parametric data and the nonparametric Wilcoxon rank sum test between young and middle-aged and elderly populations in the HC and gout groups. Pearson or Spearman correlation analysis shows that unassociated with HCs expressing Tfh1 cells **(C)**, age inversely correlated with Tfh1 cells of gout patients with a pronounced significance, *P* < 0.001 **(D)**. Age has no significant association with Tfh2 cells of HCs, *P*=0.347 **(E)**, whereas they are positively interrelated in the gout group, *P* < 0.001 **(F)**. HCs, healthy controls; ns, not significant. *P < 0.05.

To identify whether age was associated with the dysbiosis of the aforementioned gout-associated lymphocytes, individuals from both controls and cases were classified into young (age ≤44 years) and middle-aged and elderly subpopulations (age >44 years). Mean age (median (P25, P75)) was 32.00 (28.00, 38.00) years for 29 young subjects with a range of 26–44 years and was 53.00 (47.00, 57.00) for 26 middle-aged and elderly subjects with a range of 45–69 years in the healthy cohort. Mean age (median (P25, P75)) was 34.00 (28.50, 41.50) years for 21 young subjects with a range 22–44 years and was 52.00 (48.50, 61.50) for 37 middle-aged and elderly subjects with a range of 45–72 years in the gouty cohort. The ratio of young subjects and middle-aged and elderly subjects between the two cohorts was similar in statistics (*P*=0.094). Significant increased frequency of CD4^+^ T cells existed within healthy populations although there were no alterations of CD4^+^ T cells in the middle-aged and elderly populations (**Supplementary Figure 1A**). Dampened Tfh1 cells and enhanced Tfh2 cells were observed in middle-aged and elderly subjects within gout cases (**Figures 1A, B**). Tc2 cells were higher in middle-aged and elderly patients than young patients but did not change in healthy individuals (**Supplementary Figure 1C**). Other gout-related lymphocytes were found to be insignificant between the young and middle-aged and elderly groups (**Supplementary Figures 1B, D**). The association between age and Tfh1/Tfh2 cell percentage was analyzed using the Pearson or Spearman correlation test and showed a negative age association of Tfh1 cell percentage (*r*=−0.457, *p*<0.001, **Figures 1C, D**) but a positive age association of Tfh2 cell percentage in gout (*r*=0.452, *p*<0.001, **Figures 1E, F**). No significant age associations were found in the control cohort (*P*=0.399 for Tfh1, *P*=0.347 for Tfh2).

### Age-Specific Tfh2 Cell Augmentation Is Linked With Kidney Dysfunction in Gout

**Figure 2:**
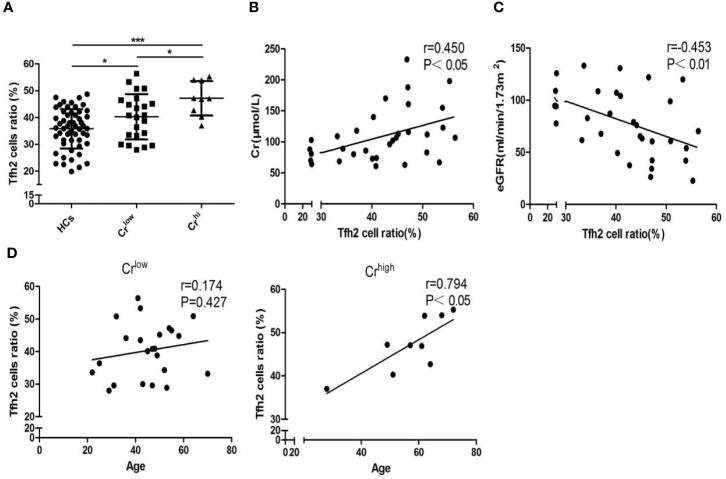
Comorbid kidney impairment linked with augmented age-specific Tfh2 cells. **(A)** Tfh2 cells of HCs, Cr^low^, and Cr^hi^ were compared based on one-ANOVO analysis followed by LSD analysis of variance. Patients have the most abundant Tfh2 cells in the Cr^hi^ group (*P*<0.05), and patients of the low Cr group are more abundant (P<0.05) when compared with controls. Furthermore, Pearson correlation analysis shows Tfh2 cells positively associated with Cr **(B)** and negatively associated with eGFR **(C)** of gout patients. **(D)** In the Cr^low^ group, age is not relevant to Tfh2 cell expression, but it has a highly positive correlation in the Cr^hi^ group. HCs, healthy controls; Cr, creatinine; eGFR, estimated glomerular filtration rate. Cr^low^ indicates those patients with Cr ≤ 116 umol/L (enzymic method). Cr^hi^ indicates those patients with Cr>116 umol/L (enzymic method) *P < 0.05. ***P < 0.001.

According to the level of serum Cr, 32 gout patients were stratified into Cr^low^ (Cr ≤ 116umol/L) and Cr^hi^ (Cr >116 umol/L) groups. More detailed results of clinical parameters, including Cr for the evaluation of kidney function, are described in **Supplementary Table 1**. Tfh2 cells were more abundant in gout patients with low Cr (*P*=0.019, **Figure 2A**) than controls and most abundant in patients with high Cr, which suggests that gout-aggravated kidney is associated with Tfh2 cell upregulation. We also observe that Tfh2 cells are positively associated with Cr (*r*=0.450, *P*< 0.05, **Figure 2B**) and inversely correlated with eGFR (*r*=0.453, *P*< 0.01, **Figure 2C**) in gout cases. To test whether age influenced kidney involvement related to Tfh2 cells in gout patients, a Pearson correlation coefficient analyzed the relation between age and Tfh2 cells in Cr^low^ and Cr^hi^ gout populations. A positive association between age and Tfh2 cells was found exclusively in Cr^hi^ gout populations (*r*=0.794, *P*<0.05, **Figure 2D**). Collectively, age-specific Tfh2 cells might have a marked impact on targeted kidney impairment.

### No Differential Distinctions Appeared in B Cell Development of Gout Patients

Mechanically, to interrogate the further pathway of Tfh cell subsets, we detected the different phases of B cells in the development process, including CD3^-^CD19^+^ total B cells, CD3^-^CD19^+^CD27^-^IgD^+^ naïve B cells, CD3^-^CD19^+^IgD^+^CD27^-^ CD38^low^CD21^low^ cells, CD3^-^CD19^+^IgD^+^IgM^+^CD27^+^CD38^+^CD24^-^ nonswitched B cells, CD3^-^CD19^+^IgD^+^IgM^+^CD27^+^CD38^+^CD24^+^ memory B cells, CD3^-^CD19^+^IgD^-^IgM^-^CD27^+^CD38^-^ class-switched memory B cells, and CD3^-^CD19^+^IgD^-^IgM^-^CD27^+^CD38^+^ plasmablasts in circulation. As a consequence, there were no novel findings in the differential distinctions between gout patients and healthy controls (**Table 2**).

**Table 2:** Differentiated alterations of B cells in gout patients.

Parameters	Marker	HCs (*n* = 56)	Gout (*n* = 58)	P value
B cells (%, median (P25, P75))	CD3-CD19^+^	10.025(6.38,12.925)	9.36(6.36,12.8)	0.522
Naïve B cells (%, median (P25, P75))	CD3-CD19^+^CD27-IgD^+^	55.7(42.9,67.85)	55.85(40.1,66.325)	0.878
Nonswitched B cells (%, median (P25, P75))	CD3-CD19^+^IgD^+^IgM^+^CD27^+^CD38^+^CD24-	0.053(0.025,0.185)	0.0685(0.0185,0.15)	0.774
Memory B cells (%, median (P25, P75))	CD3-CD19^+^IgD^+^IgM^+^CD27^+^CD38^+^CD24^+^	0.485(0.2325,1.465)	0.48(0.2,0.9975)	0.439
Class-switched B cells (%, median (P25, P75))	CD3-CD19^+^IgD-IgM-CD27^+^CD38-	7.835(4.28,9.86)	8.33(3.615,14.275)	0.520
Plasmablast (%, median (P25, P75))	CD3-CD19^+^IgD-IgM-CD27^+^CD38^+^	3.22(1.4525,8.8775)	3.75(1.3275,6.7725)	0.669

## Discussion

Overall, our study shows gout patients exhibit an overrepresentation of CD4^+^ T cells, Th2 cells, Tfh2 cells, and Tc2 cells and an underrepresentation of Tfh1 cells and Tc17 cells. Initiatively, the skewing of circulating Tfh2 cell polarization was found to be age-specific in gout patients. Furthermore, the outgrowth of age-associated Tfh2 cells contributes to gouty comorbidity with renal impairment. To the best of our knowledge, this is a novel finding to report the association of Tfh cell subgroups with gout and organ deterioration.

MSU-triggered NLRP3 activation and derived cascades of innate immune responses have been expansively confirmed in gout etiology. Meanwhile, several studies focus on specific acquired immune cells, such as RANKL-expressing T cells (5) and Th17 cells (6). However, the role of different T lymphocyte subpopulations in circulation of gout patients remains obscure. Our study shows that some specific T lymphocytes were changed in gout, including CD4^+^ T, Th2, Tfh1, Tfh2, Tc2, and Tc17, which suggests gout is associated with out-of-control growth of T lymphocytes. Follicular helper T cells, a subset of CD4^+^ T cells, are specialized regulators of T cell help to B cells and are required for induction of germinal center (GC) B cell responses (10, 11). A disproportion dominated by Tfh2 cells and Tfh17 cells has been reported in several autoimmune and allergenic diseases such as juvenile dermatomyositis (12), allergic rhinitis (13), and systemic lupus (14).

In addition, our study found the balanced skewing of gout-perturbed Tfh1/Tfh2 cells was linked with aging. Since the 1990s, accumulated human and animal experiments were conducted devoted to the impact of aging on Th1 and Th2 cells, which share the same cytokine secretions with respective Tfh1 and Tfh2 cells (6) based on total T cells or CD4^+^ T cell–produced serum cytokine detection as well as analysis at the gene or protein level. Supporting us compellingly, Abb J et al. found the production of Th1-like interferon (IFN) gamma by human PBMCs in defending against virus infection was lower in elderly subjects (greater than 50 years) than those young populations (less than 50 years) (15). Aging is accompanied by progressive decline in immune function, termed immunosenescence (16), comprising the alterations of immune cell counts and functions. Gupta S noted that molecular signaling of extrinsic and intrinsic pathways of apoptosis or programmed cell death has been proposed to explain immunosenescence (17). Cell death occurs due to apoptosis, necrosis, and autophagy. Uric acid, one of these damage-associated molecular patterns (DAMPs) (18), released by dying cells can trigger an acute inflammatory response with the stimulation of tissue-resident macrophages to produce proinflammatory mediators (19). Dying cells stimulate DC activation as an adjuvant and promote T and B cell responses when injected into animals admixed with an antigen (20, 21). In addition, immunologically active MSU crystals have been shown to stimulate the activation of DCs and augment immune responses as well (18). Mirjam Kool reported that UA crystals activated dendritic cells to promote Th2 cell immunity through spleen tyrosine kinase and PI3-kinase d signaling but independent of the inflammasome (Nlrp3, Pycard) or the interleukin-1 (Myd88, IL-1r) axis (22). From this, we postulate that the release of uric acid from dying cells that accompanies aging may contribute to the skewing polarization of Tfh2 cells in Tfh cells; the possible mechanism remains to be investigated in depth.

Gouty patients are reported to have more renal impairment than those with osteoarthritis (7). Both allopurinol and febuxostat treatments support the hypothesis that hyperuricemia per se can have an adverse impact on kidney function (23). However, to date, this conundrum regarding the cause of the strong association between impaired nephron function and gout remain to be disentangled (24). In addition, we find that the presence of kidney comorbidity is merely associated with Tfh2 cell augmentation, which provides another perspective, to some extent, that age-induced Tfh2 cells might link gout and renal comorbidity.

Prior studies validate that Tfh cells produce IL-21 to promote GC B cell proliferation, class-switch recombination, memory B cell formation, and plasma cell differentiation (25, 26). To uncover the association between Tfh cells and antigen-dependent differentiation of B cells in gout, the distribution of the circulating B cell subpopulations were observed, containing total B cells, naïve B cells, nonswitched class B cells, switched class B cells, memory B cells, and plasmablasts. However, the pronounced disproportion of these subpopulations were not observed, suggesting that Tfh2 cells might function in the pathogenesis of gout in a B cell–independent manner. As documented by Zhang et al. (27) in inflammatory bowel disease, IRF8-regulated Tfh cells can function as B cell–independent and pathogenic mediators of colitis.

## Data Availability Statement

The original contributions presented in the study are included in the article/**Supplementary Material**. Further inquiries can be directed to the corresponding author.

## Ethics Statement

The studies involving human participants were reviewed and approved by Medical Ethics Committee of the Third Affiliated Hospital of Sun Yat-sen University. Written informed consent to participate in this study was provided by the participants’ legal guardian/next of kin.

## Author Contributions

Study conceptualization: YH, XW, LG, and JG. Data curation: YH, QW, QL, BJ, YW, and SC. Formal analysis: YH, XL, JO, YJ, and YX. Methodology: YH, XW, LG, XZ, JO, QW, ZC, LF, and MQ. Project administration and resources: JG. Software: YH, JO, ZC, XZ, YJ, and LT. Supervision: JG. Writing original draft: YH, XW, LG, and JG. Writing—review and editing: YH, XW, LG, YJ, LT, ZhL, ZeL, MQ, and JG. All authors contributed to the article and approved the submitted version.

## Conflict of Interest

The authors declare that the research was conducted in the absence of any commercial or financial relationships that could be construed as a potential conflict of interest.

The handling editor declared a past co-authorship with one of the authors JG.
